# Interkingdom Complementation Reveals Structural Conservation and Functional Divergence of 14-3-3 Proteins

**DOI:** 10.1371/journal.pone.0078090

**Published:** 2013-10-11

**Authors:** Marco Lalle, Flora Leptourgidou, Serena Camerini, Edoardo Pozio, Efthimios M. C. Skoulakis

**Affiliations:** 1 Department of Infectious, Parasitic and Immunomediated Diseases, Istituto Superiore di Sanità, Rome, Italy; 2 Biomedical Sciences Research Centre “Alexander Fleming” Vari, Greece; 3 Department of Cell Biology and Neurosciences, Istituto Superiore di Sanità, Rome, Italy; Russian Academy of Sciences, Institute for Biological Instrumentation, Russian Federation

## Abstract

The 14-3-3s are small acidic cytosolic proteins that interact with multiple clients and participate in essential cellular functions in all eukaryotes. Available structural and functional information about 14-3-3s is largely derived from higher eukaryotes, which contain multiple members of this protein family suggesting functional specialization. The exceptional sequence conservation among 14-3-3 family members from diverse species suggests a common ancestor for 14-3-3s, proposed to have been similar to modern 14-3-3ε isoforms. Structural features of the sole family member from the protozoan *Giardia duodenalis* (g14-3-3), are consistent with this hypothesis, but whether g14-3-3 is functionally homologous to the epsilon isoforms is unknown. We use inter-kingdom reciprocal functional complementation and biochemical methods to determine whether g14-3-3 is structurally and functionally homologous with members of the two 14-3-3 conservation groups of the metazoan *Drosophila melanogaster*. Our results indicate that although g14-3-3 is structurally homologous to D14-3-3ε, functionally it diverges presenting characteristics of other 14-3-3s. Given the basal position of *Giardia* in eukaryotic evolution, this finding is consistent with the hypothesis that 14-3-3ε isoforms are ancestral to other family members.

## Introduction

The 14-3-3s comprise a family of small acidic polypeptides, which form dimers and are ubiquitous in all eukaryotic kingdoms [1], [2], [3]. The key feature of 14-3-3s is their ability to bind hundreds of client proteins, generally phosphorylated on a serine or threonine within short consensus binding motifs [4]. The broad 14-3-3 interaction network [5], [6], [7], [8], [9] and the essential role of these proteins may be reflected in the multiple distinct isoforms present in higher eukaryotes. The structure of both animal and plant 14-3-3s is conserved, with the monomer comprised of a bundle of nine alpha-helices (A to I) organized in a cup-like shape. All 14-3-3s contain three distinct blocks of highly invariant amino-acids [2], proposed essential for their distinct topology and functionality [10]. The most invariant block in the central region of all isoforms suggests evolution from a common ancestor before animal and plant separation [2]. Dimerization, is the elemental functional property of 14-3-3s and is mediated by interaction of largely conserved amino-acids in the amino-terminal helices A and B of one monomer with C and D of the other [11]. The identity of conserved amino-acids within these helices determines dimer stability and limits homo and hetero-dimerization options among isoforms. Dimer composition is functionally relevant because it determines the range of client proteins it interacts with.

Another remarkable characteristic of 14-3-3s is the high amino-acid sequence similarity among orthologs from different species, rather than with other isoforms of the same organism [2], [3]. This is particularly prominent among animal epsilon isoforms [3], suggesting that 14-3-3ε may be close to the common ancestral isoform. However, a central question in studying the 14-3-3 family is whether sequence conservation among orthologs also reflects functional conservation and differentiates them from other family members. We addressed this question based on the similarity of the single 14-3-3 protein of *Giardia duonenalis (syn. G. lamblia, G. intestinalis),* a worldwide parasite of the upper part of the small intestine of mammals including human [12], with the fruit fly *Drosophila melanogaster* 14-3-3s, asking for interspecies functional complementarity.

The protist genus *Giardia* belongs to the diplomonads, a group of anaerobic/microaerophilic binucleated flagellates. The highly divergent *Giardia* spp. are considered candidate early-branching eukaryotes and provide unique opportunities for gaining insights into key events of eukaryotic evolution [13], [14], [15]. In this emerging eukaryotic model, a single 14-3-3 (g14-3-3) engages an interaction network comparable to that of the yeast isoforms [5] and is critically important for parasite differentiation [16]. In contrast, the metazoan *D. melanogaster* has only two 14-3-3-encoding genes [17], but alternative splicing of the 14-3-3ζ-encoding gene *leonardo* results in three spatially restricted and apparently functionally distinct isoforms [17], [18].

In addition to alternative splicing, posttranslational modifications are utilized to increase the number of distinct isoforms temporally or cell-type-specifically, to cope with functional diversity when limited by the number of distinct 14-3-3 encoding genes in a genome [19]. Unlike the Drosophila isoforms [20], g14-3-3 is constitutively phosphorylated and stage-dependent polyglycylated, modifications essential for parasite differentiation from trophozoites into infectious cysts [21], [22]. Whether modified and unmodified g14-3-3 have distinct functions and whether they can functionally complement one or more isoforms of a higher eukaryote is currently unknown.

Because of g14-3-3s similarity to *Drosophila* and human 14-3-3ε isoforms, we investigated whether it is functionally orthologous to D14-3-3ε and conversely whether any of the fly 14-3-3 proteins function in *G. duodenalis* (henceforth referred to as *Giardia*). The two systems are ideally suited for this approach as they are highly distant evolutionarily, have well developed genetic arsenals and flies have only two 14-3-3 genes to the single protozoan counterpart. Of the three fly 14-3-3ζ (LEONARDO) isoforms, we chose LEOII because similar to D14-3-3ε it is broadly expressed spatially and temporally [18] and parallels in principle the broad distribution of g14-3-3 in *Giardia* [16], [22].

## Experimental Procedures

### Cultures and Transfection

Trophozoites of *G. duodenalis* strain WB-C6 were axenically grown and encystation induced as previously described [22]. Transgenic *Giardia* lines were generated by electroporation, selected and maintained under 100 µM puromycin (Invivogen, Toulouse, France).

*Drosophila melanogaster* were cultured as described [17]. Transgenics carrying the pUAST-*6xHis-g14-3-3* on chromosome 3 were recombined onto this chromosome carrying the *D14-3-3^ex4^* mutant allele [17] and maintained over the TM3Sb balancer. The pan-neuronal driver Elav-Gal4 (ElavG4) and the ubiquitous βTubulin-Gal4 (Tub-G4) were used to express these transgenes.

### Vector Construction

For FLAG-tagged *Giardia-*expression vectors, the FLAG epitope sequence was introduced by PCR at the 5′ end of *D14-3-3ε* and *D14-3-3ζ(leoII)* coding sequences using the primers: D14εForw:*5′* ***CCATGG*** *TGGATTATAAGGAGATGATGATAAGGGATCCACTGAGCGCGAGAACAAT-3′* (the *NcoI* site in bold and the FLAG coding sequence underlined) in combination with D14εRev: *5′-* ***GCGGCCGC*** *TTACGACACGTCCTGATC-3′* (the *NotI* site is in bold) and D14ζForw: *5′* ***CCATGG*** *TGGATTATAAGGATGATGATGATAAGGGATCCTCGACAGTCGATAAGGAAG-3′* (the *NcoI* site is in bold and the FLAG coding sequence underlined) in combination with D14ζRev: *5′* ***GCGGCCGC*** *TTAGTTGTCGCCGCCCTC-3′* (the *NotI* site is in bold). Amplified fragments were cloned in the *NcoI*/*NotI*-digested pγGFPa vector [23], replacing the GFP coding sequence under the constitutive glutamate dehydrogenase promoter to obtain plasmids “pγa-FLAG14-3-3ε” and “pγa-FLAG14-3-3ζ”. Plasmid “pγa-FLAGg14-3-3” and recombinant GST-difopein have been described previously [22].

To generate transgenes for *Drosophila*, the coding sequence of the 6XHis-g14-3-3 from plasmid pYAHis_g14-3-3 [22] was used as PCR template with the primers: g14-3-3 for: *5′GCGCCCCAGATTTTA* ***GAATTC*** *ATGGCTAGAGGA-3′* (introduced EcoRI site is in bold) and g14-3-3 rev: *5′-TCGAGTCGACCCGG* ***TCTAGA*** *CTACTACTTCTC-3′* (introduced XbaI restriction site is in bold). EcoRI/XbaI-digested PCR fragment was cloned in the EcoRI/XbaI-digested pUAST vector [24] for fly transformation and multiple independent lines were obtained.

### Preparation of Soluble Protein Fractions

Soluble *Giardia* proteins were prepared from 2×10^9^ trophozoites or encysting parasites as previously described [22].

For affinity purifications and mass spectrometry analysis, soluble proteins were obtained from 30–40 flies chilled at 4°C for 1 h, then frozen at -20°C followed by homogenization in 1 ml EB (EB, 30 mM Tris-HCl, 1 mM DTT, and 1 mM EDTA, pH 7.4), supplemented with protease and phosphatase inhibitor cocktails (Sigma), then sonicated and the lysate treated as described above.

For immunoblot and cross-linking, 1–5 whole flies were homogenized in 50 µl homogenization buffer (50 mM Tris pH 7.5, 150 mM NaCl, 1 mM EDTA, 1% Triton X–100) supplemented with a protease and phosphatase inhibitor cocktails (Sigma). Protein concentration was quantified by Quant-iT (Molecular Probes).

*Western blots*. Proteins were separated on SDS-PAGE and transferred onto PVDF membrane as described [20]. The mouse anti-FLAG mAb (Sigma-Aldrich) was used at 1∶4000, the rabbit N14 (anti-g14-3-3) [22] at 1∶5000, the rabbit polyclonal anti-14-3-3 (Ab14112, Abcam plc, Cambridge, UK) at 1∶500, the mouse AXO49 mAb at 1∶2000 [22], anti-syntaxin mAb (8C3, DSHB) at 1∶5000, anti-LEO 1∶20000 [25], anti-D14-3-3ε at 1∶2000 [17] and the monoclonal anti-His (DSHB) at 1∶2000. Appropriate HRP-conjugated secondary Abs were used at 1∶2000 and developed with the ECL system (GE Healthcare).

### Expression and Purification of Recombinant Proteins

GST and GST-Difopein were purified from *E. coli* by affinity chromatography on glutathione-sepharose 4B (GE Healthcare, Little Chalfont, England) and eluted with 10 mM glutathione (pH 8.0) as described by the manufacturer.

To purify 14-3-3 proteins from *Giardia* or *Drosophila* on GST-difopein, 3 mg of soluble proteins were incubated with 15 µg of glutathione-sepharose immobilized GST or GST-difopein in HT buffer at 4°C for 2 h. After extensive washes with HT buffer, the 14-3-3 proteins were eluted with 100 µl of 2 mM A8Ap synthetic phosphopeptide (ARAApSAPA, where pS is a phosphoserine) reproducing a 14-3-3 binding motif [26], in HT buffer. An aliquot of eluted material was run in SDS-PAGE and subjected to immunoblot as described above.

### Affinity Purifications, Pull-down and Immunoprecipitations

FLAG-tagged proteins were purified using anti-FLAG M2 mAb bound to agarose beads (Sigma-Aldrich) per manufacturer’s instructions. An equal amount of *Giardia* soluble proteins from control WB-C6 strain and transgenic lines were incubated with anti-FLAG beads at 4°C for 3 h and washed with 100 bed volumes of K-HT buffer. To selectively remove proteins bound to 14-3-3, the resin was incubated with 2 mM A8Ap synthetic phosphopeptide, at 4°C for 1 h. Finally, FLAG-fusion proteins were eluted from the resin by incubation with 200 µM synthetic FLAG-peptide at 4°C for 1 h and stored at −70°C until use.

For purification of *His-tagged protein,* 1 mg of *Drosophila* soluble proteins was incubated with 120 µl pre-cleared Ni beads (Talon) according to manufacturer, for 4 h at 4°C, followed by multiple washes in lysis buffer with 10 mM imidazole. Bead-bound proteins were recovered by addition of one volume of Laemmli buffer and heating at 95°C for 5 min.

For cross-linking experiments, 6xHis-g14-3-3 was purified from 1 mg of *Drosophila* protein lysate on Ni-beads as described above. Cross-linking was attained by incubating lysates in 3 mM BS^3^ (Pierce) as described previously [20] and then processing the samples for Western blots, or Ni affinity purification.

### Microscopy and Cell Counting

*Giardia*, trophozoites or encysting cells were prepared as described previously [22] and stained with Cy3-conjugated anti-FLAG mAb (Sigma-Aldrich), anti-g14-3-3 (N14) rabbit serum [22] revealed with Alexa-Fluor 488-conjugated anti-rabbit secondary Ab (Invitrogen, Carlsbad, CA, USA), FITC-conjugated mouse anti-CWP mAb (Waterborne Inc., New Orleans, LA, USA). Prior to microscopy using a Zeiss Axioplan microscope, cells were embedded in an anti-fading agent (Vectashield, Vector Laboratories, Burlingame, CA, USA) containing 300 nM of 4′,6-diamidino-2-phenylindole (DAPI). For image processing the Paint Shop Pro7 was used (Corel Corporation, Ottawa, Canada).

For cell counting, parasites cultured in encysting medium for 12 h were stained with Cy3-conjugated anti-FLAG mAb, FITC-conjugated anti-CWP mAb and DAPI. For each transgenic parasite line, the total amount of cells spotted on the glass-slide and positive for the anti-FLAG staining were counted. The percentage of trophozoites, encysting parasites and cysts (distinguished from trophozoites by co-staining with anti-CWP mAb) was quantified and the mean and standard error of three independent experiments calculated.

*Drosophila* tissues were fixed for 20 minutes in 4% PFA in PBS (40 mM NaH_2_PO_4_, 1 M NaCl pH 7.4), blocked for 1 hr at RT in 10% Normal Goat Serum in PBHT (20 mM PO_4_, 0.5 M NaCl, 0,2% Triton X-100, pH 7.4). Incubation with the primary antibodies anti-Elav and anti-His at (1∶50) was carried overnight at 4°C. Appropriate Alexa Fluor (Molecular Probes) secondary antibodies were used (1∶500) for 4 hr at RT. Confocal images were obtained with a Biorad Radiance 2100 system.

### Mass Spectrometry

Aliquots of affinity purified 14-3-3s (with either anti-FLAG or GST-difopein) were separated on a 1D-gel NuPAGE 4–12% (Novex, Invitrogen) run in morpholinepropanesulfoninic acid (MOPS) buffer and stained with the Colloidal Blue Staining kit (Invitrogen). Slices were excised and digested with modified sequencing-grade trypsin (Promega) according to published protocols [27]. Mass spectrometry analyses of peptide mixtures were performed with a Voyager DE-STR (Applied Biosystems) in positive reflector mode. To enhance the efficiency of phosphopeptide ionization in MALDI, 30 mg/ml of 2,5-dihydroxybenzoic acid (DHB, Sigma Aldrich) at the concentration of 30 mg/ml in 50% CH_3_CN, 1% orthophosphoric acid was used as matrix. Peptides were measured in the mass range of 750 to 4000 Da and all spectra were internally calibrated and processed via the Data Explorer software using ions coming from trypsin autodigestion of FLAG-g14-3-3.

### Sequence Analysis

Multiple alignments were performed using the Clustal W program at http://www.ebi.ac.uk/clustalw/.

## Results

### Expression and Subcellular Localization of Transgenic 14-3-3s in *G. duodenalis* and *D. melanogaster*

**Figure 1:**
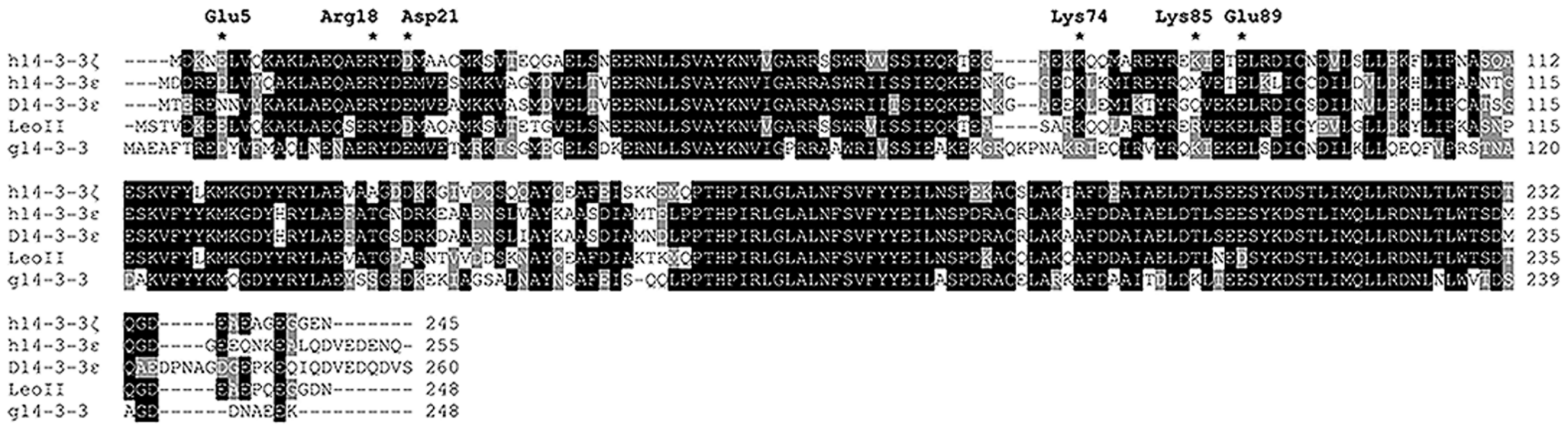
Multiple Sequence alignment of 14-3-3 proteins. *Homo sapiens* 14-3-3epsilon (h14-3-3ε, accession number P62258.) and 14-3-3zeta (h14-3-3ζ, P29312); *Drosophila melanogaster* D14-3-3epsilon (D14-3-3ε, P92177) and Leonardo II (LeoII, P29310-2); *Giardia duodenalis* 14-3-3 (g14-3-3, AAZ91664.1). Invariant or conserved amino-acids are boxed in black, those conserved in at least two proteins are highlighted in gray and divergent ones are left unboxed. Dashes indicate gaps. Stars indicate residues that in the human 14-3-3zeta form salt bridges involved in N-terminal dimerization (Arg^18^-Glu^89^, Glu^5^-Lys^74^, and Asp^21^-Lys^85^).

Sequence alignment of *Giardia* g14-3-3 with *Drosophila* D14-3-3ε and LeoII, revealed that all three proteins contain the highly invariant domains typical of all 14-3-3s [2], especially in the C-terminal region. Amino-acids unique to each of the proteins were present in the divergent regions as expected (Figure 1). Overall, g14-3-3 exhibits 61% identity and 79.8% similarity with D14-3-3ε. In contrast, g14-3-3 is 55% identical and 78.2% similar with LeoII, 55% identical and 77.5% similar with LeoI, while 54% identical and 77% similar with LeoIII (data not shown). Based on these comparisons, g14-3-3 appears homologous to D14-3-3ε, in agreement with the prediction of the ancestral 14-3-3ε hypothesis. To determine whether g14-3-3 is a functional ortholog of D14-3-3ε we took a complementary approach expressing the *Giardia* protein in *Drosophila* and the fly proteins in the protozoan.

**Figure 2:**
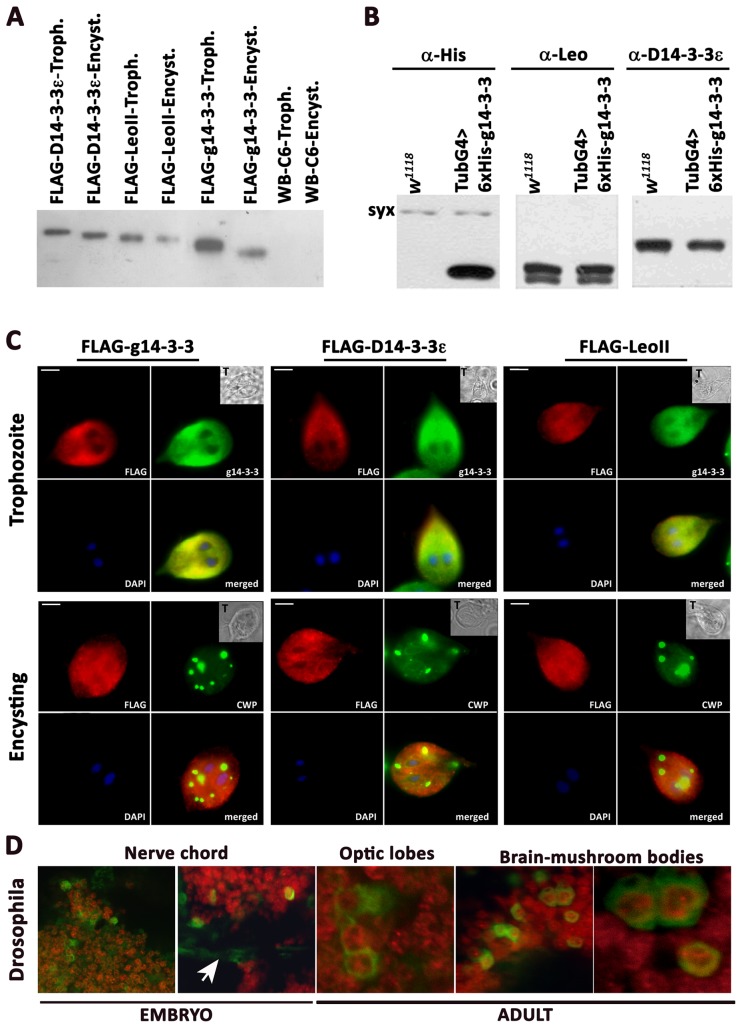
Cross species expression and subcellular localization of 14-3-3 proteins. A) Expression of *Drosophila* 14-3-3s in *Giardia*. Approximately 7 µg of proteins extracted from transfected trophozoites (T, or Troph.) and 12 hr encysting parasites (Encyst.) were separated on 12,5% SDS-PAGE, transferred onto a PVDF membrane and probed with anti-FLAG mAb. Untransfected WB-C6 and the g14-3-3 transgenic line were used as controls. B) Expression of g14-3-3 in *Drosophila*. Two adult flies per genotype were homogenized in 50µl Laemmli buffer and 10µl homogenate per lane was resolved on 12% SDS-PAGE and transferred onto a PVDF membrane and probed with the indicated antibodies. The neuronal protein Syntaxin (syx) was used to ascertain equal loading. The *w^1118^* strain, parental to the tranformants was used as control. C) Subcellular localization of the FLAG-tagged *Drosophila* 14-3-3s compared to g14-3-3-FLAG in *Giardia* trophozoites and 12h encysting parasites encysting parasite. Parasites were stained with Cy3-conjugated anti-FLAG mAb (red), rabbit anti-g14-3-3 serum (N14) followed by Alexa Fluor-488 anti-rabbit (green) or with FITC-conjugated anti-CWP mAb (green), and with DAPI (blue). Transmission light acquisition (T). Scale bars, 2.5 µm. D) g14-3-3 expression in Drosophila embryonic and adult neurons as indicated. The neuronal-specific nuclear protein Elav is red (rat anti-Elav revealed by anti-rat Alexa Fluor-555), while g14-3-3-His is green (mouse anti-His revealed by anti-mouse Alexa Fluor-488). The arrow points to an embryonic motor neuron axon which contains only the cytoplasmic His-g14-3-3. The far right panel is a magnification of the middle panel revealing g14-3-3 expression in adult mushroom body neurons.

*Drosophila* 14-3-3s were introduced in *Giardia* by cloning *D14-3-3ε* and *leoII* cDNAs [18] under the constitutive *Giardia glutamate dehydrogenase* promoter ensuring stable mid range expression in all stages, except for a small decrease during encystation [28]. A transgenic line over-expressing a FLAG-g14-3-3 was used as control. The recombinant proteins expressed in trophozoites and during encystation appeared as single species in all transfectants and absent from the parental WB-C6 strain (Figure 2A). The apparent molecular weight of g14-3-3 was larger than predicted from the primary sequence due to the previously described post-translational modifications [16]. In encysting parasites, FLAG-g14-3-3 displayed the expected decrease in apparent molecular weight consequent to the previously described shortening of the polyglycine tail occurring normally during cyst formation [22]. Intriguingly, a smaller molecular weight shift was also evident for FLAG-D14-3-3ε, suggesting modification by polyglycylation or phosphorylation. In contrast, FLAG-LeoII appeared to migrate in its predicted molecular weight irrespective of the stage of the parasites expressing it, suggesting lack of such post-translational modifications (Figure 2A).

Ubiquitous expression of transgenic 6XHis-g14-3-3 in *Drosophila* was achieved under the β-Tubulin-Gal4 (TubG4) enhancer [29] (Figure 2B). The *Giardia* protein was expressed at relatively high levels and exhibited the expected molecular weight. Importantly, the polyclonal antibodies against D14-3-3ε and Leo did not cross-react with the *Giardia* protein, which is consistent with the differences in primary sequence and facilitates differentiation between the three proteins when co-expressed. The 3 Leo species resolved into two bands in this particular gel system as reported before [20].

Because g14-3-3 polyglycylation has been linked to its nuclear localization [22], we investigated the distribution of the transgenic *Drosophila* proteins in *Giardia* trophozoites and parasites undergoing encystation. Encystation was monitored with an antibody against *Giardia* cyst wall protein (CWP) [16], [21], which decorates the encystation-specific vesicles (ESV) and in the early stage of encystation, the endoplasmic reticulum also. As previously shown [16], both anti-FLAG and anti-g14-3-3 antibodies exclusively decorated the cytoplasm in control trophozoites expressing FLAG-g14-3-3. The transgenic protein was absent from the ESVs of the encysting parasites, demonstrated by lack of co-localization with the CWP protein (Figure 2C). Similarly, FLAG-D14-3-3ε was present in the cytoplasm of trophozoites, but was not in the nuclei of encysting parasites. By contrast, FLAG-LeoII localized both in the nuclei and cytoplasm of trophozoites and encysting parasites. These immunolocalization results support the notion borne from the western analysis that FLAG-D14-3-3ε could be polyglycylated and excluded from nuclei, whereas FLAG-LeoII is not.

The subcellular localization of 6xHis-g14-3-3 under the neuronal-specific ElavG4 driver was examined in fly embryonic and adult neurons selected for their accessibility. In comparison to the nuclear Elav protein [30], 6XHis-g14-3-3 was clearly localized in the perinuclear space of the cell bodies and in axons (arrow) of adult mushroom body (MB) neurons (Figure 1D). The transgenic protein was similarly perinuclear in adult optic lobe and embryonic ventral nerve cord neurons. Similar localization was observed for non-neuronal tissues expressing 6XHis-g14-3-3 under TubG4 (not shown). Therefore, 6xHis-g14-3-3 is cytoplasmic in *Drosophila* cells as in *Giardia* trophozoites.

### Selective Dimerization of g14-3-3 with Leo

**Figure 3:**
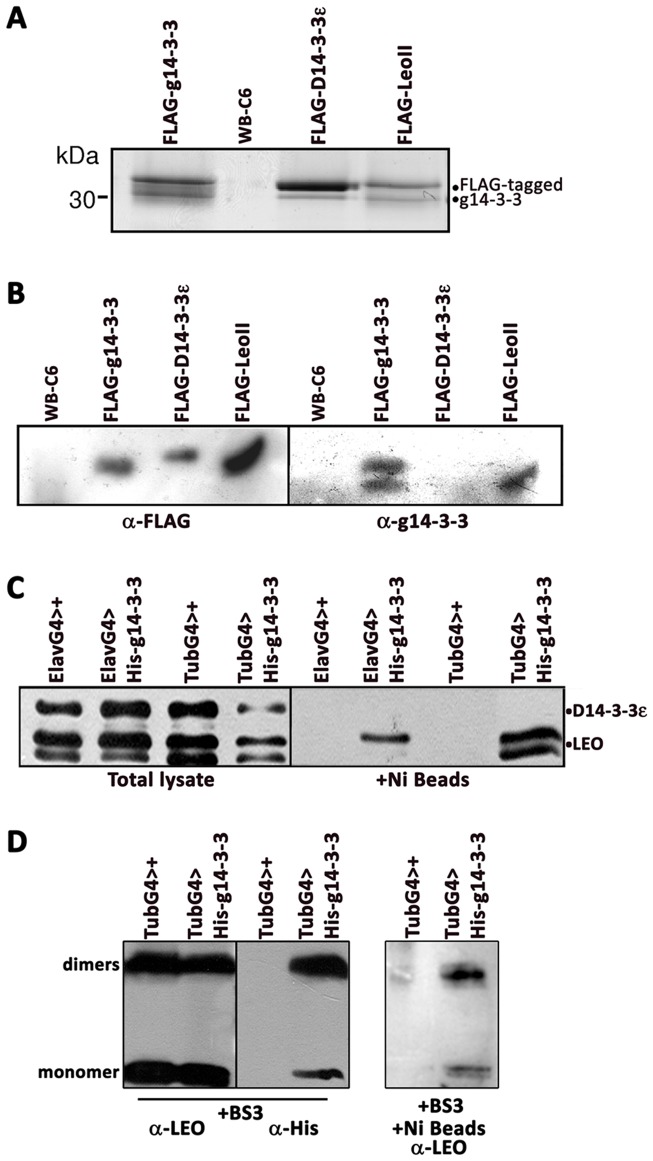
Selective heterodimerization of *Giardia* and *Drosophila* 14-3-3s. A) Coomassie stained 12% SDS-PAGE of affinity purified FLAG-tagged 14-3-3s from transfected Giardia and WB-C6 controls. The expected position of the endogenous g14-3-3 and FLAG-tagged proteins is indicated. The lower band visible in the FLAG-D14-3-3ε was identified as D14-3-3ε, representing a proteolytic product likely at N-terminus. B) Western blot analysis using 1∶10 of the affinity purified FLAG-tagged 14-3-3s separated on a 12% SDS-PAGE. The membrane was sequentially probed with anti-FLAG mAb (left panel) and then with the N14 anti-g14-3-3 serum. Untransfected WB-C6 were used as controls. C) Western blot analysis using 1∶10 of the affinity purified His-tagged g14-3-3 separated on a 12% SDS-PAGE. The transgenic protein was expressed either throughout the adult fly (TubG4) or specifically in the CNS (ElavG4). For the CNS expressed protein adult head lysates were used exclusively. The expected locations of the endogenous Drosophila proteins are indicated. Both blots were probed simultaneously with the anti-Leo and anti-D14-3-3ε antibodies. Driver alone heterozygotes (TubG4>+ or ElavG4>+) were used as controls. D) Whole fly lysates from the indicated control and His-g14-3-3 expressing animals were cross-linked with BS^3^. Complexes were separated on a 10% SDS-PAGE without (left two panels) or after His-tag affinity purification through Ni beads and the blots probed with the indicated antibodies. The membranes were probed with the indicated antibodies. The expected electrophoretic mobilities of monomers and dimers are indicated.

The default state of 14-3-3s is dimeric [10], [11], [31] and in transfected *Giardia* and transgenic *Drosophila*, 14-3-3s from different species co-exist. Because g14-3-3 is the sole isoform in *Giardia*, it normally forms homodimers. Heterodimer formation with either fly 14-3-3 would provide significant insight into their structural properties. Therefore, FLAG-tagged proteins were purified from *Giardia* trophozoites by affinity chromatography as detailed in experimental procedures. Tagged 14-3-3s were present only in the transfectant FLAG-eluted fractions, but not in controls (Figure 3A). As demonstrated already [16], endogenous g14-3-3 co-purified with FLAG-g14-3-3 (Figure 3B), but intriguingly it also co-purified with FLAG-LeoII to a comparable extent, demonstrating that the two isoforms heterodimerize. Importantly, no signal could be detected with anti-g14-3-3 in the immunopurified FLAG-D14-3-3ε fraction (Figure 3B). Although a band with molecular weight compatible with g14-3-3 was visible in the coomassie stained gel (Figure 3A), this was identified by MS analysis as D14-3-3ε (data not shown) and likely correspond to an N-terminal truncated FLAG- D14-3-3ε, since it was not detectable by the anti-FLAG antibody.

Similarly, nickel affinity chromatography of 6XHis-g14-3-3 from transgenic *Drosophila* lysates demonstrated exclusive co-purification with the endogenous Leo, but not with D14-3-3ε (Figure 3C), irrespective of whether the transgenic *Giardia* protein was expressed, only in neurons (ElavG4) or throughout the adult fly (TubG4). Interestingly, His-g14-3-3 appeared to interact with one of the Leo species resolved (Fig. 3C, lane 6), but currently its identity is under investigation. To further demonstrate the differential dimerization of 6xHis-g14-3-3 with the endogenous Leo, we chemically cross-linked the proteins with BS3, resolved the complexes by SDS-PAGE and used antibodies to probe the constitution of the dimers. The results revealed strong dimerization of Leo isoforms [20], homodimerizarion of 6xHis-g14-3-3, but also Leo/6xHis-g14-3-3 heterodimers (Figure 3D). To ascertain Leo/6xHis-g14-3-3 heterodimerization in Drosophila, we collected the complexes containing cross-linked 6xHis-g14-3-3 with nickel beads and asked whether such dimers contain Leo. Indeed, as shown in Figure 3D, His-containing dimers also co-purified with endogenous Leo, demonstrating heterodimerization of the *Giardia* and *Drosophila* proteins. These dimers did not contain detectable D14-3-3ε (data not shown). Collectively, these results suggest that g14-3-3 heterodimerizes preferentially with Leo either in *Giardia* or *Drosophila,* a property consistent with a 14-3-3ε-like protein.

### D14-3-3ε is Post-translationally Modified in *Giardia*

**Figure 4:**
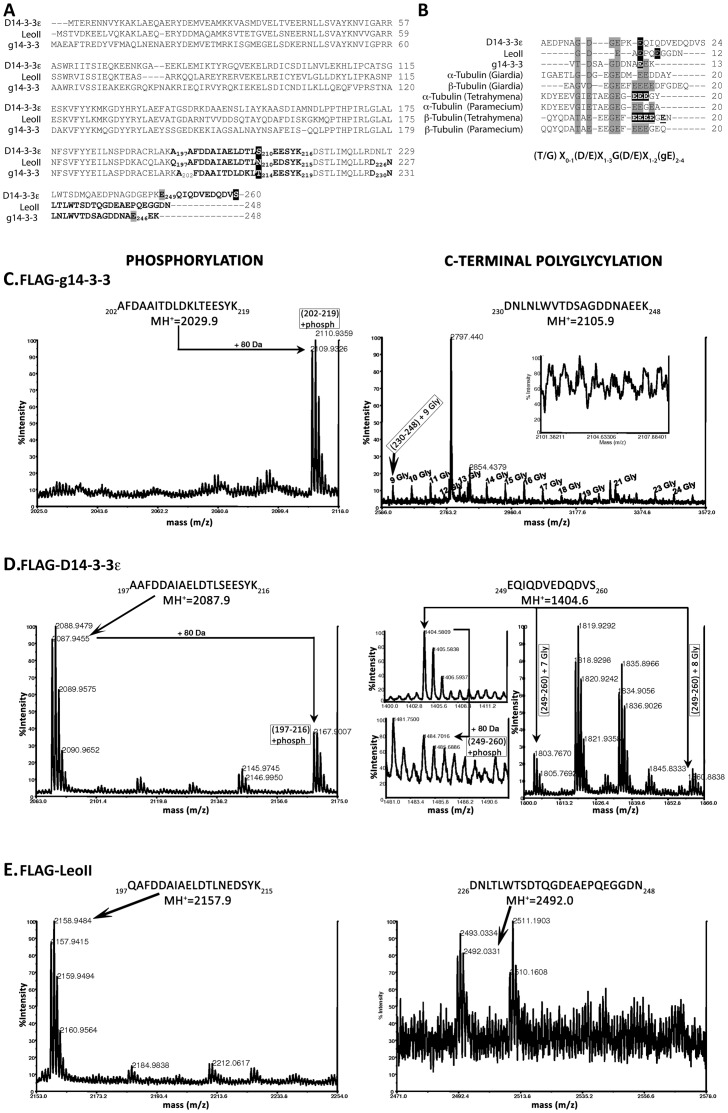
Phosophorylation and polyglycylation of 14-3-3 proteins in *Giardia*. A) Multiple Alignments of g14-3-3, LeoII and D14-3-3ε amino-acid sequences showing the peptides derived from trypsin digestion and containing the phosphorylated Thr^214^ (black boxed) and the Glu^246^ (grey boxed) of g14-3-3 and the corresponding peptides of LeoII and D14-3-3ε are in bold. Residues are numbered according to published protein sequences. B) Alignment of the C-terminus of g14-3-3, LeoII and D14-3-3ε with α- and β-tubulin of *Giardia* (GenBankTM/EBI Accession Number AAN46106 and P05304), α- and β-tubulin of *Paramecium tetraurelia* (GenBankTM/EBI Accession Number CAA67848 and CAE75646), and α- and β-tubulin of *Tetrahymena thermophila* (GenBankTM/EBI Accession Number P41351 and P41352). The alignment was performed with the ClustalW program and manually refined. The amino acids in grey define the hypothetical polyglycylation sequence [T/G]X0-1[D/E]X1-3G[D/E]X1-2[E]2-4. Experimentally defined polyglycylated glutamic acid residues are black boxed and highlighted in bold white letters. Putative polyglycylated glutamic acid residues are only black boxed. The underlined glutamic acid of *Tetrahymena* α-tubulin is predicted to be polyglycylated. C-E) MALDI-MS analysis of affinity purified FLAG-tagged transfected proteins from *Giardia* trophozoites**.** MALDI-MS spectra of FLAG-g14-3-3 (C), FLAG-D14-3-3ε (D) and FLAG-LEOII (E), encompassing the MH^+^ range of 1400–3600. Mono-isotopic masses of relevant peaks are shown. Peptides are indicated by the positions of their N- and C-termini and numbered as in the protein sequence. Peaks shifted from the theoretical MH^+^ are indicated by arrows as for the 80 Da shift due to phosphorylation. For each protein, analysis of phosphorylation is reported on the left and the analysis of C-terminal polyglycylation is reported on the right panels. C) For FLAG-g14-3-3, the calculated MH^+^ peak for peptide 202–219 at 2029.9 is clearly shifted to 2109.93 indicating phosphorylation on Thr^214^ (_202_AFDAAITDLDKL**T**EESYK_219_). On the right, the peaks corresponding to polyglycylation of the peptide (_230_DNLNLWVTDSAGDDNA**E**EK_248_) with predicted MH^+^ = 2047.92 was clearly shifted to 2105.9 indicating multiple glycines on Glu246 as revealed by their number in the lateral chain. The insert shows the lack of the peak corresponding to the unmodified 230-248 peptide. D) For D14-3-3ε-FLAG, the predicted peak at MH^+^ = 2087.9 for the peptide (_197_AAFDDAIAELDTL**S**EESYK_21_) was shifted by 80 kDa to MH^+^ = 2167.9, indicating phosphorylation at Ser^210^. On the right, the peaks corresponding to the unmodified (_249_EQIQDVEDQDVS_260_ = 1404.6 MH^+^) peptide and the shifts to higher MH^+^ consistent with addition of 7 or 8 glycines (right) and phosphorylation at Ser^260^ (+80kDa, arrow on the left) are shown. C) For LEOII-FLAG only the peaks corresponding to unmodified (_197_QAFDDAIAELDTLNEDSYK_215_ = 2157.98 MH^+^) and (_226_DNLTLWTSDTQGDEAEPQEGGDN_248_ = 2492.03 MH^+^) peptides were evident.

In *Giardia* g14-3-3 is constitutively phosphorylated at Thr^214^ and polyglycylated at Glu^246^ [21], [22]. Residues surrounding Thr^214^ of g14-3-3 are well conserved in *Drosophila* 14-3-3s (Figure 4A) as in place of Thr^214^ of g14-3-3 there is the phosphorylatable Ser^210^ in D14-3-3ε. In contrast, it is replaced with the polar uncharged Asn^210^ in LeoII. Moreover, a hypothetical polyglycylation signature sequence, [T/G]X_0-1_[D/E]X_1-3_G[D/E]X_1-2_[gE]_2-4_, where X is a polar or a negatively charged amino acid and gE is a polyglycylated glutamic acid, has been previously suggested for the g14-3-3 [22] at the C-terminus. Although as commonly observed for 14-3-3s [2], the C-termini of the three proteins diverge (Figure 4A), we searched for a similar sequence in the fly isoforms. As shown (Figure 4B), multiple alignment of the C-terminal amino acids of the D14-3-3ε and LeoII respectively, with those of g14-3-3 surrounding the polyglycylated Glu^246^ identified a polyglycylation signature sequence at the D14-3-3ε C-terminus which was clearly absent from LeoII. As reference and controls, we included in this comparison the verified polyglycylation sites of α- and β-tubulin from *G. duodenalis*, *Tetrahymena thermophila* and *Paramecium tetraurelia* [21], [22].

To ascertain that D14-3-3ε was modified in *Giardia* as convergently suggested by sequence comparison, immunofluorescence and immunoblot analysis, affinity purified FLAG-tagged proteins from trophozoites were analyzed by MALDI-MS. The mass spectrum of FLAG-g14-3-3 revealed the presence of peaks corresponding to peptide _202_AFDAAITDLDKLTEESYK_219_ (Figure 4C left panel) exclusively in the phosphorylated form at Thr^214^ (MH^+^ = 2109.96). Furthermore, inspection of the mass spectrum at high m/z values within the 2586–3572 mass range revealed the peculiar polyglycylation pattern profile (peak series at 57 Da intervals) associated with peptide _230_DNLNLWVTDSAGDDNAEEK_248_ (predicted MH^+^ = 2104.92) [21], which is consistent with the addition of up to 24 glycines (Figure 4C, right panel).

Conversely, the spectrum of FLAG-D14-3-3ε (Figure 4D, left panel) revealed two peaks, corresponding to peptide _197_AAFDDAIAELDTL**S_210_**EESYK_216_ in the un-phosphorylated (MH^+^ = 2087.9) and phosphorylated version (MH^+^ = 2167.9), suggesting that a fraction of FLAG-D14-3-3ε is phosphorylated likely on Ser^210^ corresponding to Thr^214^ in g14-3-3 (Figure 4A). However, we cannot exclude the possibility that the observed shift results from phosphorylation of either Thr^208^ or Ser^213^. Further scrutiny of the FLAG-D14-3-3ε spectra revealed the peak of unmodified C-terminal peptide _249_EQIQDVEDQDVS_260_ (MH^+^ = 1404.6), but in addition a pattern of peaks signifying addition of 7–8 glycines (Figure 4D, right panel). Sequence homology suggests polyglycylation of Glu^249^ (Figure 4A). In addition, a peak corresponding to the phosphorylated peptide 249–260 was detected (MH^+^ = 1484.6), with the only possible phosphorylation being obligatorily on Ser^260^ (Figure 4D, right panel). It is evident then, that in *Giardia*, D14-3-3ε is phosphorylated and a limited number of glycines are added at the carboxy-terminus, strongly suggesting that this *Drosophila* protein is at least in part, processed by the parasites similarly to the endogenous g14-3-3.

In contrast, tryptic digestion of FLAG-LeoII generated only the peaks corresponding to unmodified peptide _197_QAFDDAIAELDTLN_210_EDSYK_215_ with MH^+^ = 2157.98 and _226_DNLTLWTSDTQGDEAEPQEGGDN_248_ (2492.03 MH^+^) (Figure 4E, right panel), indicating that as expected, substitution of Ser^210^ with Asn^210^ abolished phosphorylation. Moreover, this result indicates that FLAG-LeoII is not modified detectably on Thr^208^ or Ser^213^ or any other residues in the vicinity, a conclusion also extended to FLAG-D14-3-3ε. Importantly, this result provides functional support for the essential role of the identified polyglycylation consensus whose absence in LeoII predicts the observed lack of modification. Therefore, D14-3-3ε is post-translationally modified in *Giardia* similarly with the endogenous g14-3-3.

### g14-3-3 is not Post-translationally Modified in *Drosophila*

**Figure 5:**
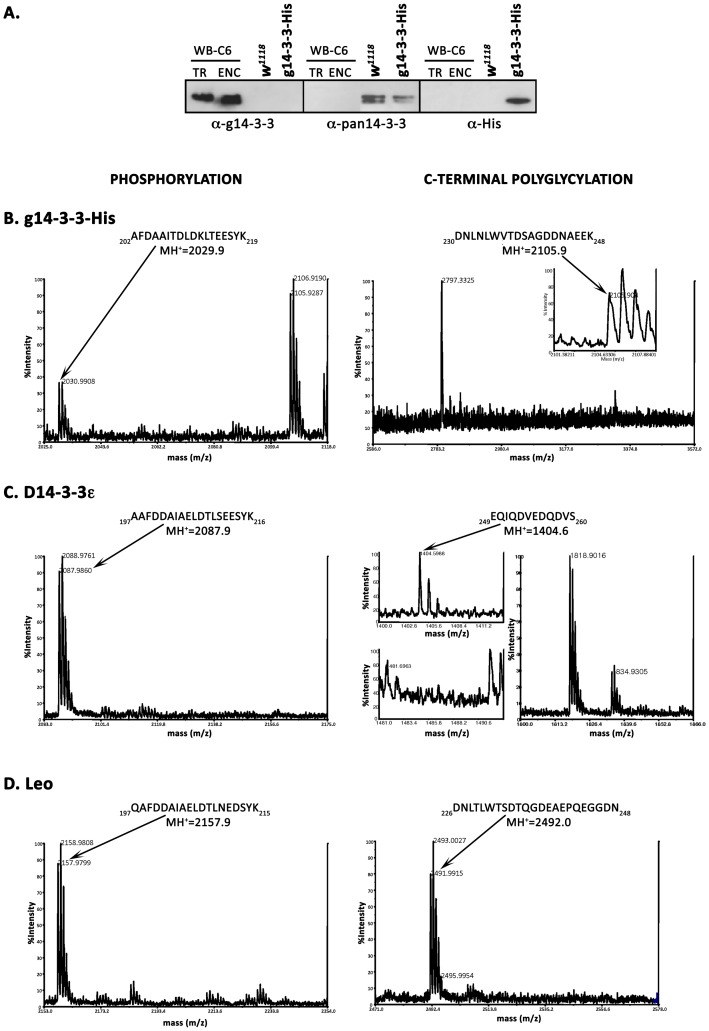
MALDI-MS analysis of affinity purified endogenous and transgenic g14-3-3 proteins from Drosophila. A) Western blot analyses of GST-difopein purified 14-3-3s (1∶10) from wild type flies and His-g14-3-3 -expressing transgenic flies and the relevant controls (WB-C6 for *Giardia* and w^1118^ for flies) were separated on 12% SDS-PAGE, blotted and the membranes probed with the indicated antibodies. The AXO49 mAb detecting the *Giardia* polyglycylated protein did not cross react with the fly isoforms, which are detected with the anti-pan14-3-3 antibody, while the transgenic g14-3-3 protein is detectable with the anti-His in fly lysates. TR denotes trophozoite lysates, while ENC indicates lysates from 12hr encysting parasites. MALDI-MS spectra of the transgenic His-g14-3-3 (B) and the endogenous Drosophila D14-3-3ε (C) and Leo (D) encompassing the MH^+^ range of 1400-3600. Mono-isotopic masses of relevant peaks are shown. Peptides are indicated by the positions of their NH_2_- and C-termini and numbered as in the protein sequence. For each protein, the analysis of phosphorylation is reported on the left panels and the analysis of C-terminal polyglycylation is reported on the right panels. Only peaks corresponding to the unmodified peptides were visible and reported here.

Given that D14-3-3ε is modified in *Giardia*, we asked whether 6xHis-g14-3-3 might also be post-transnationally modified in *Drosophila*. Therefore, whole fly lysates from controls and transgenics expressing 6xHis-g14-3-3 were purified by affinity chromatography on GST-difopein. As described before [22], polyglycylated g14-3-3 was readily purified from lysates of trophozoites and encysting parasites. Total 14-3-3 proteins were similarly purified from lysates of controls and 6xHis-g14-3-3-expressing transgenics evident as a 30 kDa doublet (Figure 5A).

The peptide mass fingerprint of proteins purified from wild type flies identified D14-3-3ε and all Leo isoforms (data not shown). The same result was obtained with proteins purified from transgenic flies, where in addition to the endogenous 14-3-3s, 6xHis-g14-3-3 was also successfully identified (data not shown). The mass spectrum of 6xHis-g14-3-3 only revealed peaks corresponding to the unmodified peptides _202_AFDAAITDLDKLTEESYK_219_ at MH^+^ = 2029.9 (Figure 5B, left) and _230_DNLNLWVTDSAGDDNAEEK_248_ at MH^+^ = 2104.92 (Figure 5B, right). Therefore, the *Giardia* protein was not post-translationally modified in *Drosophila*. Furthermore, analysis of the mass spectra of the endogenous D14-3-3ε and Leo revealed only the peaks corresponding to unmodified peptides. For D14-3-3ε (Figure 5C), the relevant peptides were _197_AAFDDAIAELDTL**S**EESYK_216_ (MH^+^ = 2087.9) and _249_EQIQDVEDQDVS_260_ (MH^+^ = 1404.6). Conversely, peptides from 14-3-3ζ were _197_QAFDDAIAELDTLNEDSYK_215_ (MH^+^ = 2157.98) and _226_DNLTLWTSDTQGDEAEPQEGGDN_248_ (MH^+^ = 2492.03) (Figure 5D). Further inspection of all spectra could not detect any other modified peptides. These results demonstrate that endogenous *Drosophila* 14-3-3s are not subjected to any major post-translational modification, at least under our experimental conditions and in the context of whole fly lysates. This is also congruent with the lack of phosphorylation or polyglycylation on the relevant peptides of the transgenic His-g14-3-3 expressed in *Drosophila*, suggesting that flies may lack the requisite enzymes.

### Negative Functional Consequences of Exogenous 14-3-3 Expression in *Giardia* and *Drosophila*

Over-expression of wild type D14-3-3ε, or Leo isoforms in *Drosophila* does not appear detrimental [17]. However, over-expression of g14-3-3 phosphorylation and polyglycylation mutants or deglycylation enzymes in *Giardia* has been reported to affect encystation [16]. Because LeoII is not post-translationally modified and D14-3-3ε presents limited polyglycylation in the parasite, we tested whether their expression may affect cyst development negatively. For each transgenic line the percentage of trophozoites, encysting parasites and cysts present in cultures after 12 hrs of growth in the encysting medium was estimated. Encysting parasites and cysts were distinguished based on expression of the CWP2 protein.

**Figure 6:**
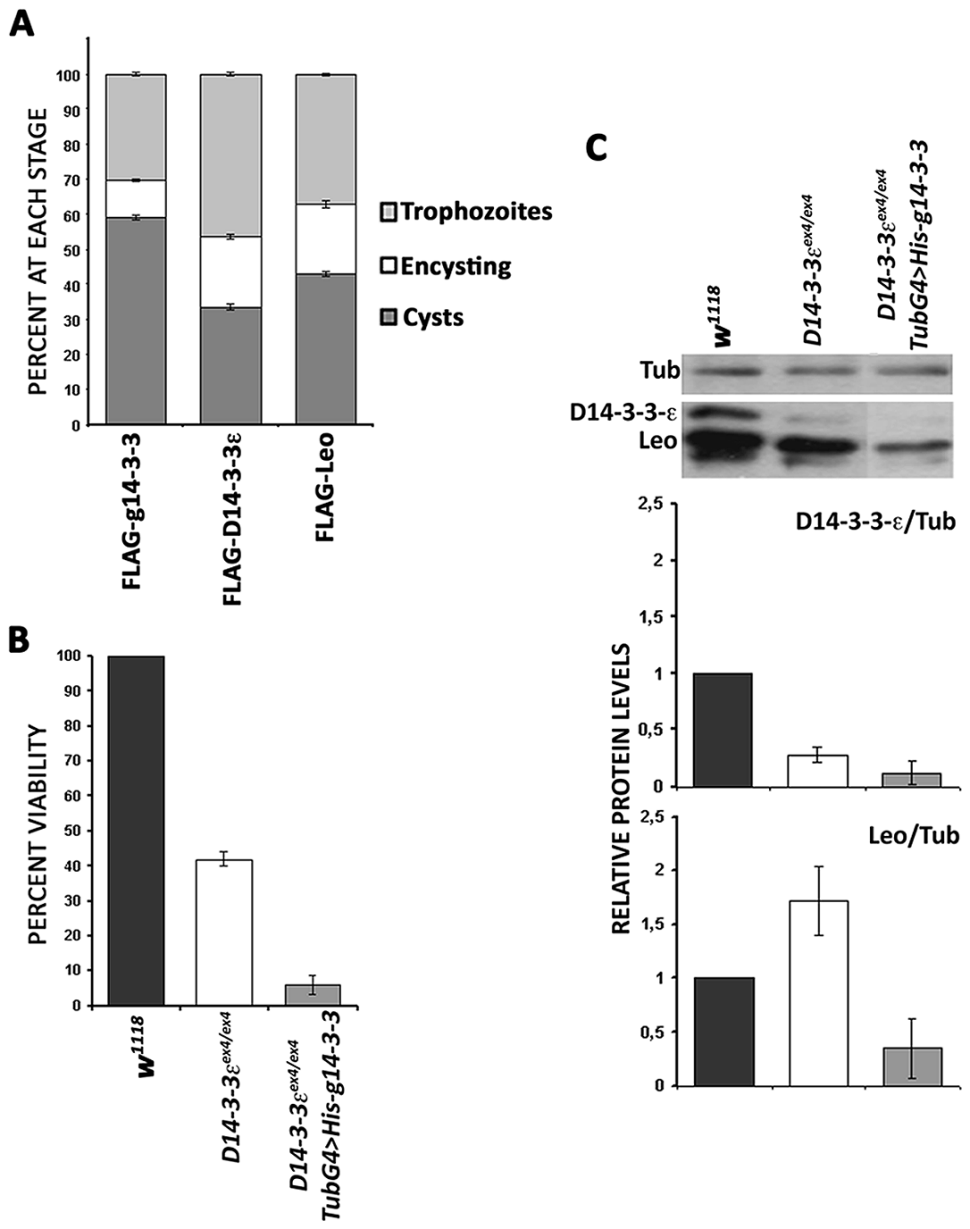
Functional consequences of exogenous 14-3-3 expression in *Giardia* and *Drosophila*. A) The number of trophozoites, encysting parasites and cysts expressing the FLAG-tagged *Drosophila* 14-3-3s were estimated by co-staining with anti-CWP and anti-FLAG antibodies after 12 h of growth in encysting medium and counting. Their numbers were compared to those of similarly cultured parasites expressing g14-3-3-FLAG. Results from three independent experiments are reported and the bars represent the total number of cells observed (approximately 1000 parasites per experiment/transfected line) and the error is represented as the standard deviation. B) The number of adult flies homozygous for the null mutation *D14-3-3ε^ex4^* alone or expressing g14-3-3 ubiquitously is reported relative to controls arbitrarily set to 100. Error bars are standard errors of the mean. C) Semi-quantitative Western blots from late embryonic extracts of the levels of endogenous Drosophila 14-3-3s in homozygotes for the *D14-3-3ε^ex4^* mutation alone or expressing g14-3-3 ubiquitously. Both anti-Leo and anti-D14-3-3ε antibodies were applied simultaneously. The level of Tubulin in the lysates was used to control for loading. The ratio of each endogenous 14-3-3 to Tub in control animals was arbitrarily set to 1 and similarly calculated ratios in the experimental genotypes are reported relative to it. The blot is representative of three in total and error bars in the graphs represent the standard errors of the mean.

Compared to *Giardia* expressing FLAG-g14-3-3 (Figure 6A), responsiveness to encystation stimuli was significantly reduced resulting in a 40% and 25% cyst reduction in transformants expressing FLAG-D14-3-3ε and FLAG-LeoII respectively. A concomitant increase in the number of encysting parasites accompanied cyst reduction and was nearly double of that observed in the FLAG-g14-3-3-expressing line. Therefore, both *Drosophila* 14-3-3s interfere with the cyst differentiation process, although probably for different reasons. Given g14-3-3 heterodimerization with LeoII and the lack of phosphorylation and polyglycylation on the latter, it is not surprising that such heterodimers may be functionally impaired for cyst formation. This is also consistent with the dominant negative effects of the g14-3-3 phosphorylation mutant T214A when co-expressed with the endogenous protein [16]. Surprisingly however, the effect seems greater upon overexpression of D14-3-3ε, which is at least in part phosphorylated and polyglycylated as is the endogenous g14-3-3. Provided that D14-3-3ε does not heterodimerize with the endogenous protein, it is likely that it competes with the endogenous g14-3-3 for relevant clients and/or modifying enzymes (such as deglycylases) impairing the encystation process.

Flies expressing 6xHis-g14-3-3 pan-neuronally under ElavG4, or ubiquitously with TubG4 seemed to develop normally and yield adults of the expected numbers without apparent morphological aberrations. Furthermore, they exhibited grossly normal behaviours and were not deficient in olfactory associative learning (data not shown). This lack of deficits prompted use of 6xHis-g14-3-3-expressing flies to complement loss of function mutations in Leo or D14-3-3ε. Loss of Leo is lethal in Drosophila and homozygotes for the null transposon mutation *leo^P1188^* are not recovered [25], [32]. Although similarly with Leo 6xHis-g14-3-3 is not post-translationally modified, its pan-neuronal or ubiquitous expression did not reverse the lethality of these and other null *leo* homozygotes (not shown). Thus, as indicated by their sequence divergence, g14-3-3 is not functionally homologous to Leo.

In contrast to *leo*, 40% of the expected *D14-3-3ε* null homozygotes are recovered (Figure 6B), because of partial compensation by up-regulation of Leo [17], [20]. Given their sequence homology and lack of post-translational modifications on 6xHis-g14-3-3 we investigated whether its expression would improve survival of *D14-3-3ε* null homozygotes (*D14-3-3ε^ex4^*). Surprisingly, pan-neuronal (not shown), or ubiquitous 6xHis-g14-3-3 expression enhanced the lethality of *D14-3-3ε^ex4^* homozygotes to nearly 100% (Figure 6B). Therefore, instead of even partial complementation of D14-3-3ε loss, g14-3-3 acted in a dominant negative manner in the null embryos.

Because 6xHis-g14-3-3 expression does not affect wild type fly development, it cannot be argued that the enhanced lethality results from interference with D14-3-3ε or Leo-interacting proteins, essential for embryonic development. An alternative explanation is that g14-3-3 interferes with, or negates the compensation by Leo over-expression in the null embryos [17]. To address this hypothesis, we quantified the levels of D14-3-3ε and Leo in late *D14-3-3ε^ex4^* homozygous embryos. Although very low levels of maternal D14-3-3ε (Acevedo and Skoulakis, unpublished) remained, Leo levels were significantly elevated in *D14-3-3ε^ex4^* null homozygotes as expected [17]. Surprisingly however, Leo levels in null homozygotes also expressing 6xHis-g14-3-3 were significantly lower than in animals not expressing the transgene (Figure 6C). These results indicate that in *Drosophila* g14-3-3 may be recognized as an endogenous 14-3-3 thereby negating the normal homeostatic response to D14-3-3ε loss [17], [20]. Because g14-3-3 does not functionally substitute for either of the two fly isoforms this results in the observed enhanced lethality of *D14-3-3ε^ex4^* nulls. This in turn confirms that the post-translational modifications of g14-3-3 are essential for the functionality of the *Giardia* protein [16], [21]-[22].

## Discussion

### g14-3-3 and D14-3-3ε are Closely Related

Our data clearly support the proposal [22] that g14-3-3 is homologous to the epsilon group of animal 14-3-3s, as it is structurally and functionally closest to D14-3-3ε of the two classes (ε and ζ) of fly 14-3-3s [33]. Because *Giardia* is suggested to represent an early branching eukaryote [15], our results are congruent with the notion that 14-3-3ε represents the member of the 14-3-3 family more similar to the ancestral protein which may have given rise to other isoforms by duplication and divergence.

As for the endogenous protein, polyglycylation appears sufficient to exclude D14-3-3ε from the nuclei of *Giardia* trophozoites, in agreement with the suggested role of the polyglycine chain in preventing g14-3-3 nucleo-cytoplasmic shuttling. In contrast, D14-3-3ε was not found in the nuclei of encysting parasites despite the presence of a short polyglycine chain. This suggests that polyglycine chain length may be critically important for nuclear import factor interaction and its reduced length on D14-3-3ε prohibits translocation. Alternatively, putative import factors are required in addition to length-independent polyglycine chain recognition and they fail to recognize features on D14-3-3ε conducive to nuclear localization. In fact, only a fraction of D14-3-3ε is phosphorylated in the peptide Ala_197_-Lys_216_ and phosphorylation of this region has been proven critical for g14-3-3 function, at least during encystation [16]. The inefficient phosphorylation and polyglycylation of D14-3-3ε in *Giardia*, functionally confirms the importance of its sequence divergence surrounding the post-translational modification sites on g14-3-3. In agreement with this notion, LeoII (and LeoI and LeoIII) being more divergent in these sequences is not modified at all and as the polyglycylation defective E246A g14-3-3 mutant [16], appears in *Giardia* nuclei stage-independently. Moreover, the post-translational modifications on g14-3-3 and the consequent changes in subcellular localization may reflect an adaptation similar to that used by *Drosophila* to increase the functional 14-3-3ζ isoforms by tissue specific alternative splicing.

Because preferential g14-3-3/LeoII heterodimerization occurs in both *Drosophila* and *Giardia*, post-translational modifications are not required or affect the process. Interestingly, although g14-3-3 must be homodimeric in *Giardia* with such homodimers also observed in *Drosophila*, it heterodimerizes preferentially with LeoII in both systems. Because D14-3-3ε/g14-3-3 heterodimers were not detected in *Giardia* or *Drosophila*, g14-3-3 homodimers and LeoII/g14-3-3 heterodimers are likely more stable and hence favoured over D14-3-3ε/g14-3-3. Homodimerization of human 14-3-3ζ is primarily mediated by three salt bridges, Arg^18^-Glu^89^, Glu^5^-Lys^74^, and Asp^21^-Lys^85^ (Liu et al., 1995), and the corresponding residues are partially conserved in LeoII (Arg^21^-Glu^92^, Glu^8^-Arg^77^, and Asp^24^-Arg^88^). In human 14-3-3ε and in D14-3-3ε, only the first salt bridge can be formed (corresponding to Arg^19^-Glu^92^ in D14-3-3ε), whereas the other residues are substituted in D14-3-3ε by Asn^6^-Glu^77^ and Glu^22^-Gln^88^. Intriguingly, despite the overall sequence homology with the epsilon subgroup, homodimerization of g14-3-3 may be stabilized by three salt bridges formed between Arg^22^-Glu^97^, Asp^9^-Lys^82^, and Glu^25^-Lys^93^ (Fiorillo et al., in preparation), similarly to 14-3-3ζ. Consistent with our observations then, LeoII/g14-3-3 heterodimers may be stabilized by gAsp^9^-dArg^77^, gGlu^25^-dArg^88^, gArg^22^-dGlu^92^ (where g indicates *Giardia* and d *Drosophila*) salt bridges. In contrast, the D14-3-3ε/g14-3-3 heterodimer can be stabilized only by the lone salt bridge, gArg^22^-dGlu^92^, whereas gAsp^9^-dGlu^77^ may cause charge repulsion between the two molecules. Because g14-3-3 can form multiple salt bridges presumably to stabilize its own homodimers in a manner akin to 14-3-3ζ, it appears to possess features of both epsilon and zeta isoforms. The later is consistent with the notion that *Giardia* may be a derived rather than primitive organism.

### g14-3-3 is not Functionally Homologous to D14-3-3ε

The kinase responsible for Thr^214^ phosphorylation of g14-3-3 in the parasite is currently unknown. Hence we cannot determine whether failure to phosphorylate g14-3-3 in flies is due to absence of a functional homolog. However, g14-3-3 polyglycylation in *Giardia* is performed by the bifunctional enzyme gTTLL3, a member of the tubulin tyrosine ligase-like family [21]. Interestingly, *Drosophila* possesses polyglycylated microtubules [34], and 7 distinct genes encoding putative TTLL proteins (Flybase), two of which have been annotated as DmTTLL3A and DmTTLL3B [35]. In *Drosophila*, DmTTLL3A mono- and polyglycylates the α- and the β-tubulin, whereas DmTTLL3B mono- and polyglycylates non-tubulin proteins [35].

It is unlikely that lack of D14-3-3ε and g14-3-3 polyglycylation in *Drosophila* results from distinct spatial distributions with DmTTLL3s. D14-3-3ε is present in all *Drosophila* developmental stages and tissues tested [17], [25] and at least for the gonads, it is co-expressed with DmTTLL3A [35], [36]. At least DmTTLL3A should be co-expressed with 6xHis-g14-3-3 under the ubiquitous TubGal4 driver [36]. It is likely then that the sequence recognized on g14-3-3 and D14-3-3ε by gTTLL3 is not a polyglycylation site for the *Drosophila* enzymes. Because polyglycylation is not evident in the fly 14-3-3s, this mechanism of g14-3-3 compartmentalization and stage-specific functions in *Giardia* does not seem to be utilized in *Drosophila*. Therefore, a major functional modification of g14-3-3 in the parasite is absent from its *Drosophila* homolog.

Our date indicate that despite the similarity of the two proteins, g14-3-3 is not a functional ortholog of D14-3-3ε, at least for processes essential for embryonic viability. The dominant negative effect of g14-3-3 expression in *D14-3-3ε* nulls is probably a consequence of its recognition as an endogenous 14-3-3 protein by the cellular mechanisms responsible for 14-3-3 homeostasis [17], [20]. Because g14-3-3 heterodimerizes with Leo, it is likely that the presence of Leo heterodimers, which are absent in *D14-3-3ε* nulls, negate the homeostatic response. The lack of post-translational modifications and/or aberrant folding potentially rendering g14-3-3 non-functional in *Drosophila*, suggest that g14-3-3/Leo heterodimers may also contribute to the dominant negative effects of the *Giardia* protein in the fly embryos.

In conclusion, our data indicate that despite the high level of sequence conservation and structural homology among eukaryotic 14-3-3 proteins, functionality and specificity likely reside in post-translational modifications and particular amino-acids as previously suggested [17], [18]. We propose that the single g14-3-3 from the early branching but highly specialized protozoan *Giardia*, may represent an ancestral 14-3-3 containing features common to different subgroups of 14-3-3 and that gene duplication and divergence events have subdivided specific 14-3-3 functions, residing in single of a few amino-acid changes, giving rise to the multimember 14-3-3 families in vertebrates and plants.
